# Crystalline Structure-Dependent Mechanical and Thermoelectric Performance in Ag_2_Se_1‐*x*_S_*x*_ System

**DOI:** 10.34133/2020/6591981

**Published:** 2020-07-31

**Authors:** Jiasheng Liang, Pengfei Qiu, Yuan Zhu, Hui Huang, Zhiqiang Gao, Zhen Zhang, Xun Shi, Lidong Chen

**Affiliations:** ^1^State Key Laboratory of High Performance Ceramics and Superfine Microstructure, Shanghai Institute of Ceramics, Chinese Academy of Sciences, Shanghai 200050, China; ^2^Center of Materials Science and Optoelectronics Engineering, University of Chinese Academy of Sciences, Beijing 100049, China; ^3^Division of Solid-State Electronics, Department of Electrical Engineering, Uppsala University, Uppsala, Sweden; ^4^School of Physical Science and Technology, ShanghaiTech University, Shanghai 201210, China

## Abstract

Self-powered wearable electronics require thermoelectric materials simultaneously with a high dimensionless figure of merit (*zT*) and good flexibility to convert the heat discharged by the human body into electricity. Ag_2_(S,Se)-based semiconducting materials can well satisfy these requirements, and thus, they are attracting great attention in thermoelectric society recently. Ag_2_(S,Se) crystalizes in an orthorhombic structure or monoclinic structure, depending on the detailed S/Se atomic ratio, but the relationship between its crystalline structure and mechanical/thermoelectric performance is still unclear to date. In this study, a series of Ag_2_Se_1‐*x*_S_*x*_ (*x* = 0, 0.1, 0.2, 0.3, 0.4, and 0.45) samples were prepared and their mechanical and thermoelectric performance dependence on the crystalline structure was systematically investigated. *x* = 0.3 in the Ag_2_Se_1‐*x*_S_*x*_ system was found to be the transition boundary between orthorhombic and monoclinic structures. Mechanical property measurement shows that the orthorhombic Ag_2_Se_1‐*x*_S_*x*_ samples are brittle while the monoclinic Ag_2_Se_1‐*x*_S*_x_* samples are ductile and flexible. In addition, the orthorhombic Ag_2_Se_1‐*x*_S_*x*_ samples show better electrical transport performance and higher *zT* than the monoclinic samples under a comparable carrier concentration, most likely due to their weaker electron-phonon interactions. This study sheds light on the further development of flexible inorganic TE materials.

## 1. Introduction

Recently, thermoelectric (TE) technology shows a great potential to be used as a sustainable power source in wearable electronics [1–3]. Via harvesting the heat discharged by the human body and converting it into electricity, the wearable electronics using TE technology can be self-powered without using any external batteries. The energy conversion efficiency of a TE material is determined by the dimensionless figure of merit, *zT* = *α*^2^*σT*/*κ*, where *α* is the Seebeck coefficient, *σ* is the electrical conductivity, *κ* is the thermal conductivity, and *T* is the absolute temperature. The TE material used in wearable electronics should possess high *zT* to maximize the energy conversion efficiency and good flexibility to match the curved surface of skin and endure repeated bending during service [4–6].

**Figure 1:**
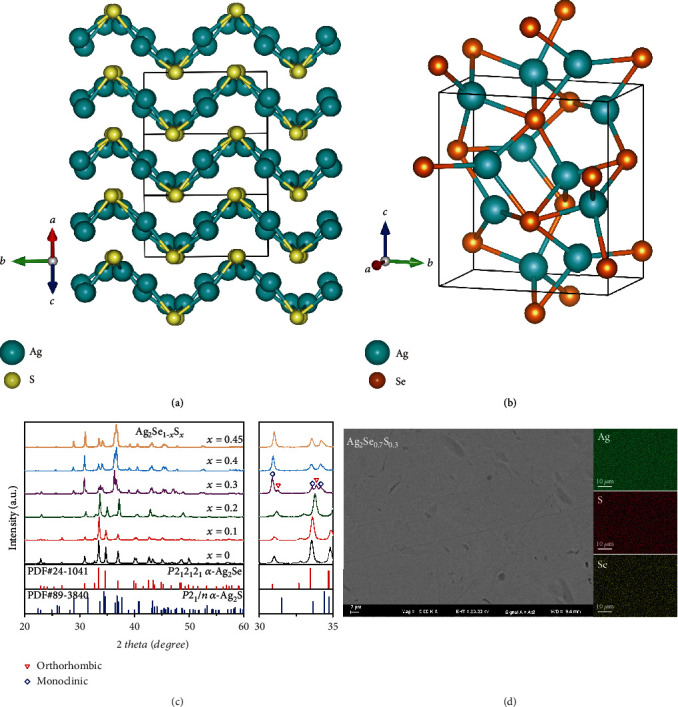
Crystalline structures for (a) monoclinic Ag_2_S and (b) orthorhombic Ag_2_Se. (c) Room-temperature X-ray diffraction patterns for Ag_2_Se_1‐*x*_S_*x*_ (*x* = 0, 0.1, 0.2, 0.3, 0.4, and 0.45) samples. The right panel shows the magnification around 2*θ* = 30 ~ 35°. (d) Backscatter electron (AsB) image and elemental energy dispersive spectroscopy (EDS) mappings of Ag_2_Se_0.7_S_0.3_.

To date, Bi_2_Te_3_-based alloys are still the best room-temperature TE materials, but their application in wearable electronics is limited due to their inherent brittleness [7–14]. In contrast, the organic TE polymers show good flexibility, but their *zT*s are low [15–20]. Recently, we reported a class of Ag_2_S-based flexible inorganic TE materials with excellent intrinsic flexibility and high *zT*s [21]. Monoclinic Ag_2_S shows surprisingly high ductility at room temperature due to its wrinkled layer structure (space group *P*2_1_/*n*, with the sketch map shown in Figure 1(a), and weak Ag-S chemical bonds [22, 23]. However, its relatively wide band gap (around 1 eV) yields low *σ* only in the order of 10^−1^ S m^−1^. As a result, *zT* of the stoichiometric Ag_2_S around room temperature is very poor. Alloying Se or/and Te in Ag_2_S can significantly improve *σ* and enhance *zT* to 0.26 for Ag_2_S_0.5_Se_0.5_ and 0.44 for Ag_2_S_0.5_Se_0.45_Te_0.05_ at 300 K. Particularly, previous study showed that the good ductility can be well maintained when the Se alloying content in Ag_2_S reaches 50% or the Te alloying content reaches 20%, enabling these materials very suitable to be used in flexible wearable electronics. The above results arouse great interest on the Ag_2_S-based materials in TE society [21, 23–25].

Being different with ductile Ag_2_S, Ag_2_Se is a brittle material. It adopts an orthorhombic structure (space group *P*2_1_2_1_2_1_) with the sketch map shown in Figure 1(b). The band gap of orthorhombic Ag_2_Se is around 0.2 eV, about one-fifth of that of monoclinic Ag_2_S. Orthorhombic Ag_2_Se has very high carrier mobility (~10^3^ cm^−2^ V^−1^ S^−1^), excellent *σ* (~10^5^ S m^−1^), and extremely low lattice thermal conductivity (~0.3-0.5 W m^−1^ K^−1^), resulting in high *zT* about 0.4~1.0 at 300 K [26–32]. Despite the different crystalline structures of Ag_2_S and Ag_2_Se, Bindi and Pingitore [33] reported that Ag_2_S and Ag_2_Se can form a continuous solid solution. The room-temperature crystalline structure of Ag_2_S_1‐*x*_Se_*x*_ solid solution is the same with the monoclinic Ag_2_S when *x* ≤ 0.6, but the same with the orthorhombic Ag_2_Se when *x* ≥ 0.7. Considering that the carrier concentration of Ag_2_Se is about several orders of magnitude higher than that of Ag_2_S [22], Ag_2_S_1‐*x*_Se_*x*_ solid solution might process continuous adjustable carrier concentrations and TE properties. The mechanical properties and TE performance of Ag_2_Se_1‐*x*_S_*x*_ (0.5 ≤ *x* ≤ 1) have been already reported previously [33], but those of Ag_2_Se_1‐*x*_S_*x*_ (0 ≤ *x* ≤ 0.5) have not been investigated yet. Particularly, the relationship between crystalline structure and mechanical/TE performance for Ag_2_Se_1‐*x*_S_*x*_ solid solution is still unclear so far.

In this study, a series of polycrystalline Ag_2_Se_1‐*x*_S_*x*_ (*x* = 0, 0.1, 0.2, 0.3, 0.4, and 0.45) samples were prepared. Their crystalline structures, mechanical properties, and TE properties were systematically investigated. *x* = 0.3 in the Ag_2_Se_1‐*x*_S_*x*_ system was identified to be the transition boundary of the orthorhombic structure and monoclinic structure. Orthorhombic Ag_2_Se_1‐*x*_S_*x*_ (*x* = 0, 0.1, and 0.2) samples are brittle, while monoclinic Ag_2_Se_1‐*x*_S_*x*_ (*x* = 0.4 and 0.45) are ductile and flexible. Due to the stronger electron-phonon interaction, monoclinic Ag_2_Se_0.6_S_0.4_ has lower PF and *zT* than orthorhombic Ag_2_Se_1‐*x*_S_*x*_ (*x* = 0, 0.1, and 0.2). However, the superior TE performance and thermal stability to the organic TE materials and the intrinsically good flexibility still promise a great potential for monoclinic Ag_2_Se_1‐*x*_S_*x*_ to be used in wearable electronics.

## 2. Results and Discussion

Figure 1(c) shows the X-ray diffraction patterns for the prepared Ag_2_Se_1‐*x*_S_*x*_ (*x* = 0, 0.1, 0.2, 0.3, 0.4, and 0.45) obtained at room temperature. When the *S* content *x* < 0.3, the diffraction peaks are well indexed to the orthorhombic structure of Ag_2_Se with the space group of *P*2_1_2_1_2_1_. This indicates that Ag_2_Se_0.9_S_0.1_ and Ag_2_Se_0.8_S_0.2_ still adopt the same crystalline structure with Ag_2_Se at room temperature. When *x* = 0.4 and 0.45, the diffraction peaks agree well with the monoclinic structure of Ag_2_S phase with the space group of *P*2_1_/*n*. Likewise, the Ag_2_Se_1‐*x*_S_*x*_ (*x* ≥ 0.5) samples also crystalize in the same monoclinic structure of Ag_2_S [21]. Elemental energy dispersive spectroscopy (EDS) mappings confirm that all elements are homogeneously distributed in Ag_2_Se_0.9_S_0.1_, Ag_2_Se_0.8_S_0.2_, Ag_2_Se_0.6_S_0.4_, and Ag_2_Se_0.55_S_0.45_. No obvious secondary phase is observed. Thus, these samples are phase pure. Likewise, scanning electron microscopy (SEM) performed on the cross-section of Ag_2_Se_0.6_S_0.4_ indicates that it has an obvious layered structure (cf. Fig. S1).

One special composition in the Ag_2_Se_1‐*x*_S_*x*_ system is Ag_2_Se_0.7_S_0.3_. As shown in Figure 1(c), its X-ray diffraction pattern looks like that of monoclinic Ag_2_Se_0.6_S_0.4_. However, besides those belonging to the monoclinic structure, some extra diffraction peaks with weak intensities are also observed, such as (102) at 2*θ* = 31° and (112) at 2*θ* = 34° (right panel in Figure 1(c)). These are indexed to the orthorhombic structure. Thus, Ag_2_Se_0.7_S_0.3_ is believed to be a mixture rather than a pure phase, in which the main phase crystalizes in the monoclinic structure while the secondary phase crystalizes in the orthorhombic structure. This result is different from that proposed by Bindi and Pingitore [33] that Ag_2_Se_0.7_S_0.3_ crystalizes in the same orthorhombic structure with Ag_2_Se. Figure 1(d) shows the EDS mappings performed on Ag_2_Se_0.7_S_0.3_. Surprisingly, all elements are still homogeneously distributed. Thus, it is concluded that the two different phases in Ag_2_Se_0.7_S_0.3_ have very similar chemical compositions but different crystalline structures. A similar polymorphic feature has been also observed in the Cu_2_Se_1‐*x*_S_*x*_ system [34]. Combining the above result, it can be concluded that the transition boundary between the monoclinic and orthorhombic structures in Ag_2_S_1‐*x*_Se_*x*_ solid solution should be around *x* = 0.3.

**Figure 2:**
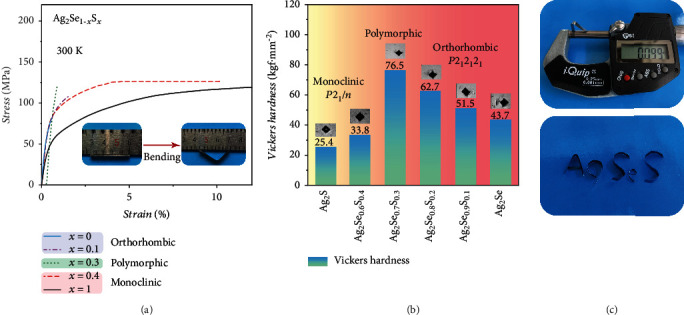
(a) Strain-stress curves for the three-point bending test of Ag_2_Se_1‐*x*_S_*x*_ (*x* = 0, 0.1, 0.3, and 0.4) samples at 300 K. The tests were performed by using a dynamic mechanical analyzer (DMA). The insets show the optical images of Ag_2_Se_0.6_S_0.4_ sample before and after the bending test. The data for Ag_2_S are included for comparison [22]. (b) Vickers hardness of Ag_2_Se_1‐*x*_S_*x*_ under the load of 0.3 kgf. The insets are micrographs of indention. Vickers hardness of Ag_2_S reported in Ref. 22 is included for comparison. (c) Ag_2_Se_0.6_S_0.4_ strips twisted into various shapes. The thickness of Ag_2_Se_0.6_S_0.4_ strips is 0.1 mm.

Ag_2_Se_1‐*x*_S_*x*_ (*x* = 0, 0.1, 0.2, 0.3, 0.4, and 0.45) samples possess structure-dependent mechanical properties. The three-point bending test and Vickers hardness test were performed on Ag_2_Se_1‐*x*_S_*x*_. The material with larger bending deformation or small Vickers hardness is usually a benefit for the application in flexible electronics [2, 3, 6].  Figure 2(a) shows that orthorhombic Ag_2_Se can only endure a very small bending strain before cracking. The maximum bending deformation is about 0.56%. Likewise, orthorhombic Ag_2_Se_0.9_S_0.1_ also possesses a maximum bending deformation around 1.5%. The case is different for monoclinic Ag_2_Se_0.6_S_0.4_, which exhibits a bending deformation above 10% without cracking. Previously, large bending deformation was also observed for monoclinic Ag_2_Se_1‐*x*_S_*x*_ (*x* = 0.5, 0.7, 0.9, and 1) in the bending test. It should be noted that such deformation is plastic, which is different from the elastic deformation of brittle materials with low dimension. Likewise, Vickers hardness for orthorhombic Ag_2_Se is 43.7 Hv at room temperature. It is monotonously increased to 51.5 Hv and 62.7 Hv for the orthorhombic Ag_2_Se_0.9_S_0.1_ and Ag_2_Se_0.8_S_0.2_, respectively. However, monoclinic Ag_2_Se_0.6_S_0.4_ has low Vickers hardness of only 33.8 Hv, about half of that for orthorhombic Ag_2_Se_0.8_S_0.2_. As shown in Figure 2(c), Ag_2_Se_0.6_S_0.4_ can be directly cut into thin strips with thickness about 0.1 mm like metal. Furthermore, these metal-like strips can be twisted into various shapes without cracking, confirming that Ag_2_Se_0.6_S_0.4_ has good ductility and flexibility.

The above test results prove that the monoclinic structure has significantly better ductility and flexibility in the Ag_2_Se_1‐*x*_S_*x*_ system. The high cleavage energy and low slipping energy, related to the unique wrinkled layer structure, are the fundamental reason for the good ductility observed in monoclinic Ag_2_Se_1‐*x*_S_*x*_. Previous investigation found that the S atoms in monoclinic Ag_2_S always bonded to part of the surrounding Ag atoms during slipping [22, 35], which is benefit for preventing the material's cleavage under mechanical stress. Likewise, it enables the bonding energy between Ag and S atoms to change fluently, yielding a low energy barrier for slipping. However, in the orthorhombic structure, such unique wrinkled layer structure does not exist; thus, the orthorhombic Ag_2_Se_1‐*x*_S_*x*_ samples are brittle. Here, it should be noted that although the main phase in Ag_2_Se_0.7_S_0.3_ crystalizes in the monoclinic structure, Ag_2_Se_0.7_S_0.3_ still has low maximum bending deformation around 0.88%. The reason should be attributed to the presence of brittle orthorhombic grains among the ductile monoclinic grains. The second phase strengthening effect caused by these brittle orthorhombic grains will impede the movement of atoms, dislocations, or interfaces under mechanical stress like that in the pure orthorhombic phase, yielding poor ductility for Ag_2_Se_0.7_S_0.3_.

**Figure 3:**
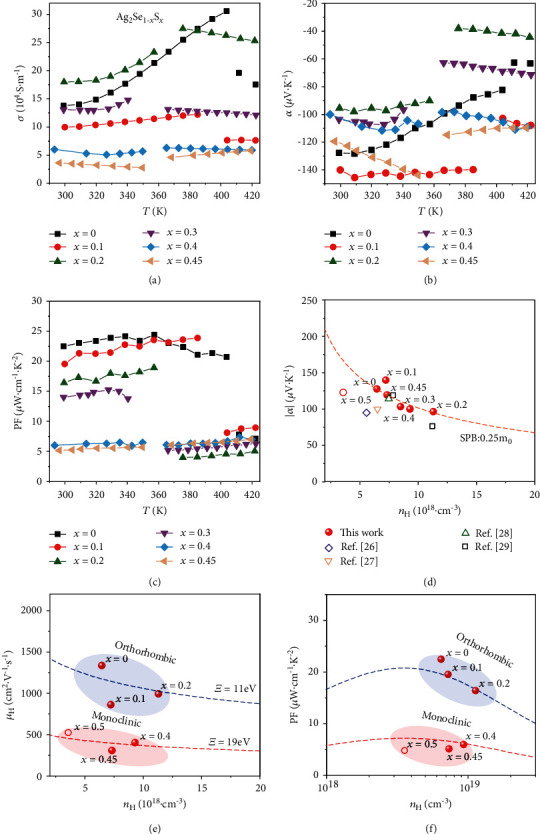
Temperature dependences of (a) electrical conductivity *σ* and (b) Seebeck coefficient *α* for Ag_2_Se_1‐*x*_S_*x*_ (*x* = 0, 0.1, 0.2, 0.3, 0.4, and 0.45). (c) Temperature dependence of power factor (PF) for Ag_2_Se_1‐*x*_S_*x*_ (*x* = 0, 0.1, 0.2, 0.3, 0.4, and 0.45). (d) Seebeck coefficient *α*, (e) Hall carrier mobility *μ*_H_, and (f) PF as a function of carrier concentration *n*_H_ for Ag_2_Se_1‐*x*_S_*x*_ at 300 K. The dashed lines represent the theoretical curves based on the single parabolic band (SPB) model with a dominated scattering mechanism by acoustic phonons. The data for Ag_2_Se and Ag_2_Se_0.5_S_0.5_ reported before are added for comparison [21, 26–29].

Figure 3 shows the measured *σ* and *α* for Ag_2_Se_1‐*x*_S_*x*_ (*x* = 0, 0.1, 0.2, 0.3, 0.4, and 0.45). All samples possess negative *α*, indicating that they are *n*-type semiconductors with electrons dominating the electrical transports. This scenario is the same with those Ag_2_Se_1‐*x*_S_*x*_ (*x* ≥ 0.5) samples. Obvious *σ* and *α* discontinuous jumps are observed between 360 K and 420 K, which are attributed to the orthorhombic-cubic or monoclinic-cubic phase transition. For example, Ag_2_Se experiences orthorhombic-cubic phase transition around 410 K. At 300 K, *σ* for Ag_2_Se is about 1.4 × 10^5^ S·m^−1^. It increases with increasing temperature, reaching around 3.1 × 10^5^ S·m^−1^ at 403 K, and then decreases to 1.9 × 10^5^ S·m^−1^ after the phase transition. The *σ* value for Ag_2_Se_0.55_S_0.45_ at 300 K is only 3.6 × 10^4^ S·m^−1^, almost one-fourth of the pristine Ag_2_Se. Likewise, the *α* values vary from -146 *μ*V·K^−1^ to -105 *μ*V·K^−1^ at 300 K and from -45 *μ*V·K^−1^ to -109 *μ*V·K^−1^ at 420 K, without obvious chemical composition dependence. Furthermore, Fig. S2 shows that the prepared samples have good reproducibility. When the chemical composition and fabrication process are fixed, different batches of samples have comparable *σ* and *α* data.

With the purpose to understand the electrical transport properties of Ag_2_Se_1‐*x*_S_*x*_, Hall measurements were performed. The Hall carrier concentration (*n*_H_) and mobility (*μ*_H_) at 300 K are listed in Table SI. All Ag_2_Se_1‐*x*_S_*x*_ samples possess *n*_H_ in the order of 10^18^ cm^−3^. However, being similar with the above measured *σ* and *α*, the *n*_H_ values do not have monotonous variation with the sulfur content. The different contents of intrinsic defects inside the lattice, such as Ag interstitials, are expected to be the reason for this scenario. Likewise, the *μ*_H_ values for the Ag_2_Se_1‐*x*_S_*x*_ samples are in the range of 309 cm^2^·V^−1^·s^−1^ to 1337 cm^2^·V^−1^·s^−1^. These *μ*_H_ values are quite high as compared with other state-of-the-art TE materials, such as 190 cm^2^·V^−1^·s^−1^ for *n*-type Bi_2_Te_3_ [36] and 48 cm^2^·V^−1^·s^−1^ for *n*-type filled skutterudites [37]. A single parabolic model (SPB) is used to further understand the electrical transports of Ag_2_Se_1‐*x*_S_*x*_. According to the SPB model, the Seebeck coefficient, carrier concentration, and carrier mobility can be correlated as follows [38–40]:

(1) $$\begin{array}{c} \alpha =\frac{\kappa_{\text{B}}}{e}\left[\frac{\left(2+\lambda \right)F_{\lambda +1}\left(\eta \right)}{\left(1+\lambda \right)F_{\lambda }\left(\eta \right)}-\eta \right], \end{array}$$

(2) $$\begin{array}{c} n_{\text{H}}=4\pi {\left(\frac{2m^{*}k_{\text{B}}T}{h^{2}}\right)}^{3/2}\frac{F_{1/2}\left(\eta \right)}{r_{\text{H}}}, \end{array}$$

(3) $$\begin{array}{c} \mu_{\text{H}}=\frac{3\sqrt{\pi }}{4}\frac{\left(1/2\right)+2\lambda }{1+\lambda }\frac{1}{\Gamma \left(2+\lambda \right)}\frac{F_{2\lambda -1/2}}{F_{\lambda }}\mu_{\text{ph}}, \end{array}$$

(4) $$\begin{array}{c} \mu_{\text{ph}}=\frac{{\left(8\pi \right)}^{1/2}{e\hbar }^{4}{{\rho v}_{\text{l}}}^{2}}{3{\left(k_{\text{B}}T\right)}^{3/2}{\left(m^{*}\right)}^{5/2}\Xi^{2}}, \end{array}$$

(5) $$\begin{array}{c} r_{\text{H}}=\frac{3}{4}\frac{F_{1/2}\left(\eta \right)F_{-1/2}\left(\eta \right)}{{F_{0}}^{2}\left(\eta \right)}, \end{array}$$

where  *k*_B_ is the Boltzmann constant, *λ* is the scattering factor, *e* is the electron charge, *m*^∗^ is the density-of-state effective mass, *r*_H_ is the Hall factor, *ρ* is the sample density, *v*_l_ is the velocity of longitudinal sound waves, *μ*_ph_ is the drift mobility for acoustic phonon scattering in the nondegenerate limit, and *Ξ* is the deformation potential. *F*_*m*_(*η*) is the Fermi integrals, and it is given by equation *F*_*m*_(*η*) = ∫_0_^∞^*x*^*m*^*dx*/(1 + *e*^*x*−*η*^), where *x* represents the reduced carrier energy and *η* = *E*_*F*_/*k*_B_*T* is the reduced Fermi energy.

Figure 3(d) presents that the *α* and *n*_H_ data for the present Ag_2_Se_1‐*x*_S_*x*_ (*x* = 0, 0.1, 0.2, 0.3, 0.4, and 0.45) samples roughly fall around the calculated theoretical Pisarenko curve with *m*^∗^ = 0.25*m*_e_ (*m*_e_ is the mass of free electron) and *λ* = 0 (acoustic phonon scattering). For comparison, the previously reported data for Ag_2_Se and Ag_2_Se_0.5_S_0.5_ are also summarized in Figure 3(d). These data also fall around the theoretical curve. This scenario suggests that all these samples might possess a similar band structure near the Fermi level, despite the fact that some of them crystalize in the monoclinic structure while others in the orthorhombic structure. This is possible because the conduction band minimums of Ag_2_S and Ag_2_Se are at the same gamma point and both of them are mainly dominated by Ag-5s electrons [21, 41]. Alloying S at the Se sites would mainly alter the valence band maximum instead of the conduction band minimum, yielding the similar *m*^∗^ mentioned above. In addition, the small *m*^∗^, about 0.25*m*_e_ for these Ag_2_(S,Se) samples, are responsible for their higher *μ*_H_ than those for *n*-type Bi_2_Te_3_ [36] and filled skutterudites [37] mentioned above.

Figure 3(e) plots the *μ*_H_ and *n*_H_ relationship at 300 K for Ag_2_Se_1‐*x*_S_*x*_ (*x* = 0, 0.1, 0.2, 0.4, 0.45, and 0.5). The data for Ag_2_Se_0.7_S_0.3_ are not included because its polymorphic feature might introduce extra boundary scattering to electrons and interrupt the understanding on the carrier mobility. As shown in Figure 3(e), under the similar *n*_H_, the Ag_2_Se_1‐*x*_S_*x*_ (*x* = 0, 0.1, and 0.2) samples with an orthorhombic structure have higher *μ*_H_ than the Ag_2_Se_1‐*x*_S_*x*_ (*x* = 0.4, 0.45, and 0.5) samples with a monoclinic structure. By fitting the experimental data of the *μ*_H_ and *n*_H_ relationship using Equations (1)–(5), deformation potential values of 11 eV and 19 eV can be extracted for the orthorhombic Ag_2_Se_1‐*x*_S_*x*_ (*x* = 0, 0.1, and 0.2) and the monoclinic Ag_2_Se_1‐*x*_S_*x*_ (0.4, 0.45, and 0.5), respectively. The thermal vibrations of the crystal lattice can influence the energy-band structure and perturb the carrier transports. The intensity of such interaction between electrons and phonons can be reflected by the magnitude of *Ξ* [42]. The lower *Ξ* value for the orthorhombic Ag_2_Se_1‐*x*_S_*x*_ suggests that the electron-phonon interaction wherein is weaker than that in the monoclinic Ag_2_Se_1‐*x*_S_*x*_. This is responsible for the higher *μ*_H_ observed for the orthorhombic Ag_2_Se_1‐*x*_S_*x*_.

The power factors (PF) for Ag_2_Se_1‐*x*_S_*x*_ (*x* = 0, 0.1, 0.2, 0.3, 0.4, and 0.45) samples calculated from the formula PF = *α*^2^ · *σ* are shown in Figure 3(c). At 300 K, the PF value for Ag_2_Se reaches 22.5 *μ*W·cm^−1^·K^−2^. It gradually decreases with increasing the S-alloying content. At 300 K, the PF for Ag_2_Se_0.55_S_0.45_ is just 5.2 *μ*W·cm^−1^·K^−2^, about one-fourth of that for Ag_2_Se. The greatly reduced *σ* is responsible for the lowered PF. The PF value for Ag_2_Se_0.6_S_0.4_ is comparable with that for Ag_2_Se_0.5_S_0.5_ reported before [21, 24], about 4.8 *μ*W·cm^−1^·K^−2^ at 300 K. Based on the SPB model, the theoretical PF vs. *n*_H_ curves at 300 K can be obtained for the orthorhombic and monoclinic Ag_2_Se_1‐*x*_S_*x*_ samples, respectively. As shown in Figure 3(f), the experimental PF and *n*_H_ data basically fall around these lines. Under the comparable *n*_H_, the PF for the orthorhombic Ag_2_Se_1‐*x*_S_*x*_ samples are much higher than those of the monoclinic Ag_2_Se_1‐*x*_S_*x*_ samples. The reason is that the orthorhombic samples have weaker electron-phonon interaction than the monoclinic samples, which yields lower *Ξ* value for higher *μ*_H_ and PF. In addition, based on the SPB model, the optimal carrier concentration (*n*_opt,PF_) corresponding to the peak PF is around 3‐4 × 10^18^ cm^−3^ for both orthorhombic and monoclinic Ag_2_Se_1‐*x*_S_*x*_. *n*_H_ for the present orthorhombic Ag_2_Se_1‐*x*_S_*x*_ (*x* = 0, 0.1, and 0.2) and monoclinic Ag_2_Se_1‐*x*_S_*x*_ (*x* = 0.4) samples are already close to this *n*_opt,PF_ value.

**Figure 4:**
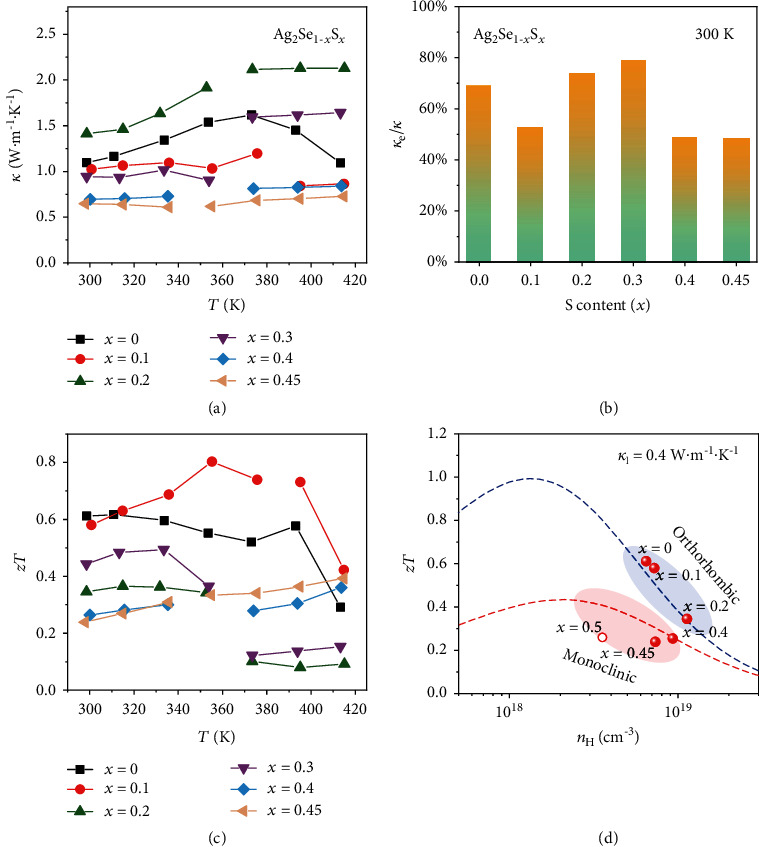
(a) Temperature dependence of thermal conductivity for Ag_2_Se_1‐*x*_S_*x*_ (*x* = 0, 0.1, 0.2, 0.3, 0.4, and 0.45). (b) The ratio of electronic thermal conductivity to thermal conductivity (*κ*_e_/*κ*) at 300 K for Ag_2_Se_1‐*x*_S_*x*_. (c) Temperature dependence of TE figure of merit *zT* for Ag_2_Se_1‐*x*_S_*x*_ (*x* = 0, 0.1, 0.2, 0.3, 0.4, and 0.45). (d) *zT* as a function of carrier concentration *n*_H_ for Ag_2_Se_1‐*x*_S_*x*_ at 300 K. The dashed lines represent the theoretical curves based on the single parabolic band (SPB) model. The data for Ag_2_Se_0.5_S_0.5_ reported before are added for comparison.

Figure 4(a) shows *κ* as a function of temperature for Ag_2_Se_1‐*x*_S_*x*_ (*x* = 0, 0.1, 0.2, 0.3, 0.4, and 0.45). The discontinuous jumps on the *κ* curves are attributed to the orthorhombic-cubic or monoclinic-cubic phase transitions. *κ* ranges from 0.7 W·m^−1^·K^−1^ to 1.4 W·m^−1^·K^−1^ for all samples. Ag_2_Se_0.6_S_0.4_ exhibits the lowest *κ* among all samples. *κ* for Ag_2_Se_0.6_S_0.4_ at 300 K is around 0.7 W·m^−1^·K^−1^. This value is about 36% decrement as compared with that for Ag_2_Se_0.8_S_0.2_. Generally, *κ* is composed of two parts: the electron part *κ*_e_ and the lattice part *κ*_L_. *κ*_e_ can be calculated according to the Wiedemann-Franz law [43]. Figure 4(b) shows the calculated *κ*_e_/*κ* for Ag_2_Se_1‐*x*_S_*x*_ (*x* = 0, 0.1, 0.2, 0.3, 0.4, and 0.45). It can be seen that the *κ*_e_/*κ* values are in the range of 48% to 80%, indicating that the carriers' contribution is very important in the thermal transports. By subtracting *κ*_e_ from *κ*, *κ*_L_ can be obtained. The results at 300 K are listed in Table SI. They range from 0.20 W·m^−1^·K^−1^ to 0.48 W·m^−1^·K^−1^, which are very low values as compared with those for the state-of-the-art TE materials [44–49]. The proximity to the cubic superionic phase is responsible for the low *κ*_L_ values observed in these orthorhombic and monoclinic phases [23].

Based on the measured *α*, *σ*, and *κ*, *zT* can be calculated according to *zT* = *α*^2^*σT*/*κ*.*zT* for orthorhombic Ag_2_Se is around 0.6 in the whole measured temperature range, which is comparable with those reported before [28–30]. Orthorhombic Ag_2_Se_0.9_S_0.1_ and Ag_2_Se_0.8_S_0.2_ possess comparable *zTs* with Ag_2_Se. However, *zTs* for monoclinic Ag_2_Se_0.6_S_0.4_ and Ag_2_Se_0.55_S_0.45_ are much lower than those for orthorhombic Ag_2_Se. At 300 K, *zT* is just 0.26 for Ag_2_Se_0.6_S_0.4_ and 0.23 for Ag_2_Se_0.55_S_0.45_.

As shown in Figure 4(d), the theoretical *zT* vs. *n*_H_ curves for orthorhombic and monoclinic Ag_2_Se_1‐*x*_S_*x*_ samples at 300 K are given by assuming that all samples possess the same *κ*_L_ = 0.4 W m^−1^ K^−1^. The experimental *zT* and *n*_H_ data basically fall around these lines. Under the comparable *n*_H_, *zT* for the orthorhombic Ag_2_Se_1‐*x*_S_*x*_ samples is much higher than that of the monoclinic Ag_2_Se_1‐*x*_S_*x*_ samples because their weaker electron-phonon interaction yields lower *Ξ* value and larger *μ*_H_ for higher PF. The optimal carrier concentration (*n*_opt,*zT*_) corresponding to the peak *zT* is around 1‐2 × 10^18^ cm^−3^ for orthorhombic Ag_2_Se_1‐*x*_S_*x*_, while 2‐3 × 10^18^ cm^−3^ for monoclinic Ag_2_Se_1‐*x*_S_*x*_. *n*_H_ for the present orthorhombic Ag_2_Se_1‐*x*_S_*x*_ (*x* = 0, 0.1, and 0.2) and monoclinic Ag_2_Se_1‐*x*_S_*x*_ (*x* = 0.4, 0.45, and 0.5) samples are still higher than these *n*_opt,*zT*_ values. Thus, if their carrier concentration can be further reduced, higher *zT* can be expected.

**Figure 5:**
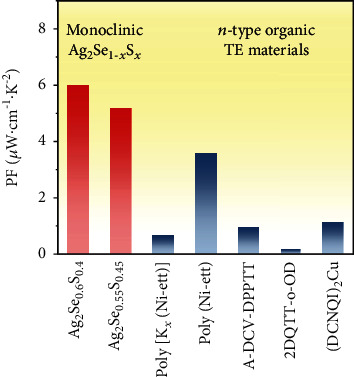
Comparisons of power factor values for *n*-type monoclinic Ag_2_Se_1‐*x*_S_*x*_ and *n*-type organic TE materials reported before [16, 19, 51–53].

Although the TE performances for ductile monoclinic Ag_2_Se_1‐*x*_S_*x*_ are lower than those for brittle orthorhombic Ag_2_Se_1‐*x*_S_*x*_, their performances are still much higher than those for the organic TE materials reported before. This can be clearly reflected by the scenario shown in Figure 5. The PF values for most of the *n*-type TE organic TE materials are lower than 1 *μ*W·cm^−1^·K^−2^ at 300 K. Even for the best *n*-type TE organic TE materials reported so far, poly(Ni-ett) [19], its PF, 3.6 *μ*W·cm^−1^·K^−2^ at 300 K, is still lower than the present monoclinic Ag_2_Se_1‐*x*_S_*x*_. The superior PF values achieved in the monoclinic Ag_2_Se_1‐*x*_S_*x*_ samples would promise high power output when they are fabricated into TE devices. In addition, most *n*-type organic TE materials are unstable in the air because their electron affinity is too low to stabilize the *n*-type dopants [2, 50]. In contrast, the present ductile monoclinic Ag_2_Se_1‐*x*_S_*x*_ samples are inert to oxygen or water; thus, they can realize good service stability in real application. Combining the intrinsically good flexibility and ductility, these monoclinic Ag_2_Se_1‐*x*_S_*x*_ samples show great potential to be used in wearable electronics.

## 3. Conclusion

In summary, this study systematically studied the crystalline structure, mechanical properties, and TE properties of Ag_2_Se_1‐*x*_S_*x*_ (*x* = 0, 0.1, 0.2, 0.3, 0.4, and 0.45). Ag_2_Se_1‐*x*_S_*x*_ samples crystalize in the orthorhombic structure when *x* ≤ 0.2, while in the monoclinic structure when *x* ≥ 0.4. Only the monoclinic Ag_2_Se_1‐*x*_S_*x*_ samples possess good ductility and flexibility. Under comparable carrier concentration range, the orthorhombic Ag_2_Se_1‐*x*_S_*x*_ samples have higher carrier mobility, larger power factor, and better *zT* than the monoclinic samples because they have weaker electron-phonon interaction. This leads to lower *zT* values of monoclinic Ag_2_Se_1‐*x*_S_*x*_ (*x* = 0.4 and 0.45) samples than those of the orthorhombic Ag_2_Se_1‐*x*_S_*x*_ (*x* = 0.1 and 0.2) samples. However, the higher PF and better thermal stability promise a great potential for monoclinic Ag_2_Se_1‐*x*_S_*x*_ to be used in wearable electronics.

## Supplementary Materials

Supplementary Materials Materials and methods. Fig. S1: cross-section image of Ag_2_Se_0.6_S_0.4_ bulk sample. Fig. S2: electrical properties for three batches of Ag_2_Se_0.8_S_0.2_ and Ag_2_Se_0.6_S_0.4_. Table SI: room-temperature thermoelectric properties of Ag_2_Se_1‐*x*_S_*x*_ samples.
