# Supplementary material for: Crystalline Structure-Dependent Mechanical and Thermoelectric Performance in Ag2Se1‐xSx System
Source: Research (Wash D C). 2020 Jul 31;2020:6591981. doi: 10.34133/2020/6591981 (PMC7521025; doi:10.34133/2020/6591981)
Supplement: Supplementary Materials — Materials and methods. Fig. S1: cross-section image of Ag2Se0.6S0.4 bulk sample. Fig. S2: electrical properties for three batches of Ag2Se0.8S0.2 and Ag2Se0.6S0.4. Table SI: room-temperature thermoelectric properties of Ag2Se1‐xSx samples. [file 6591981.f1.docx]

**Supplemental Material**

**Crystalline structure dependent mechanical and thermoelectric performance in** **Ag_2_Se_1-_*_x_*S*_x_* system**

Jiasheng Liang ^a,b^, Pengfei Qiu^a*^, Yuan Zhu^c^, Hui Huang ^a,b^, Zhiqiang Gao ^a,b,d^, Zhen Zhang^c^, Xun Shi^a*^, and Lidong Chen^a,b^

^a^State Key Laboratory of High Performance Ceramics and Superfine Microstructure, Shanghai Institute of Ceramics, Chinese Academy of Sciences, Shanghai 200050, China.

^b^Center of Materials Science and Optoelectronics Engineering, University of Chinese Academy of Sciences, Beijing 100049, China.

^c^Division of Solid-State Electronics, Department of Electrical Engineering, Uppsala University, Uppsala, Sweden.

^d^School of Physical Science and Technology, ShanghaiTech University, Shanghai 201210, China

## Materials & methods

Sample Synthesis. Polycrystalline Ag_2_Se_1-_*_x_*S*_x_* (*x* = 0, 0.1, 0.2, 0.3, 0.4, and 0.45) samples were prepared from high purity elements Ag (99.999%, Alfa Aesar, shots), S (99.999%, Alfa Aesar, powders), and Se (99.999%, Alfa Aesar, shots). The mixture was weighed out according to the chemistry stoichiometry, loaded into carbon-coated quartz tube, and sealed in vacuum. The sealed quartz tubes were heated to 1273 K, dwelt for 12 hours, and then slowly furnace-cooled to room temperature within 60 hours. After that, the tube was annealed for 3 days at 723 K to obtain the final product. The ingot was crashed into powders. Then, spark plasma sintering process was conducted at 623 K for 5 min under a pressure of 60 MPa to obtain dense bulks.

Sample Characterization. The phase composition of the samples was examined by X-ray diffraction (XRD) analysis (D8 ADVANCE instrument, Bruker Co. Ltd). The microstructure was characterized by scanning electron microscopy (SEM) instrument (ZEISS® Supra 55) equipped with an energy dispersive spectrometer (EDS, Horiba 250). Electrical conductivity and Seebeck coefficient were measured on bulk samples with approximate dimensions of 2 × 2 × 7.5 mm^3^, using the modified thermal expansion equipment (Netzsch, DIL 402C) from 300 K up to 420 K. The thermal conductivity was calculated from the formula *κ= DC_p_d*, where the thermal diffusivity (*D*) was measured by laser ﬂash system (Netzsch® LFA 457) under argon atmosphere, the specific heat capacity (*C_p_*) was estimated by Dulong-Petit law, and the density (*d*) was measured by using the Archimedes method. Hall coefficient (*R_H_*) was measured in a Hall measurement system (LakeShore® 8400 series) by sweeping the magnetic field from -0.9 T to +0.9 T. Hall carrier concentration (*n_H_*) and Hall mobility (*μ_H_*) were estimated by the relations *n_H_* *= 1/eR_H_* and *μ_H_ = σR_H_*, respectively. A polished disc with a thickness of about 1.5 mm was used to measure the Vickers hardness (TUKON-2100B, Instron). Bending tests on the bulk specimens were conducted on a dynamic mechanical analyzer (DMA) with loading rate of 0.11N/min. The specimen cross section for bending test was about 1.2 × 3 mm^2^, and the span length is fixed at 20 mm.





**Fig.S1** Cross-section image of Ag_2_Se_0.6_S_0.4_ bulk sample. The cross section was obtained by bending a 0.1mm thick Ag_2_Se_0.6_S_0.4_ strip which has been dipped in liquid nitrogen for 10 minutes.


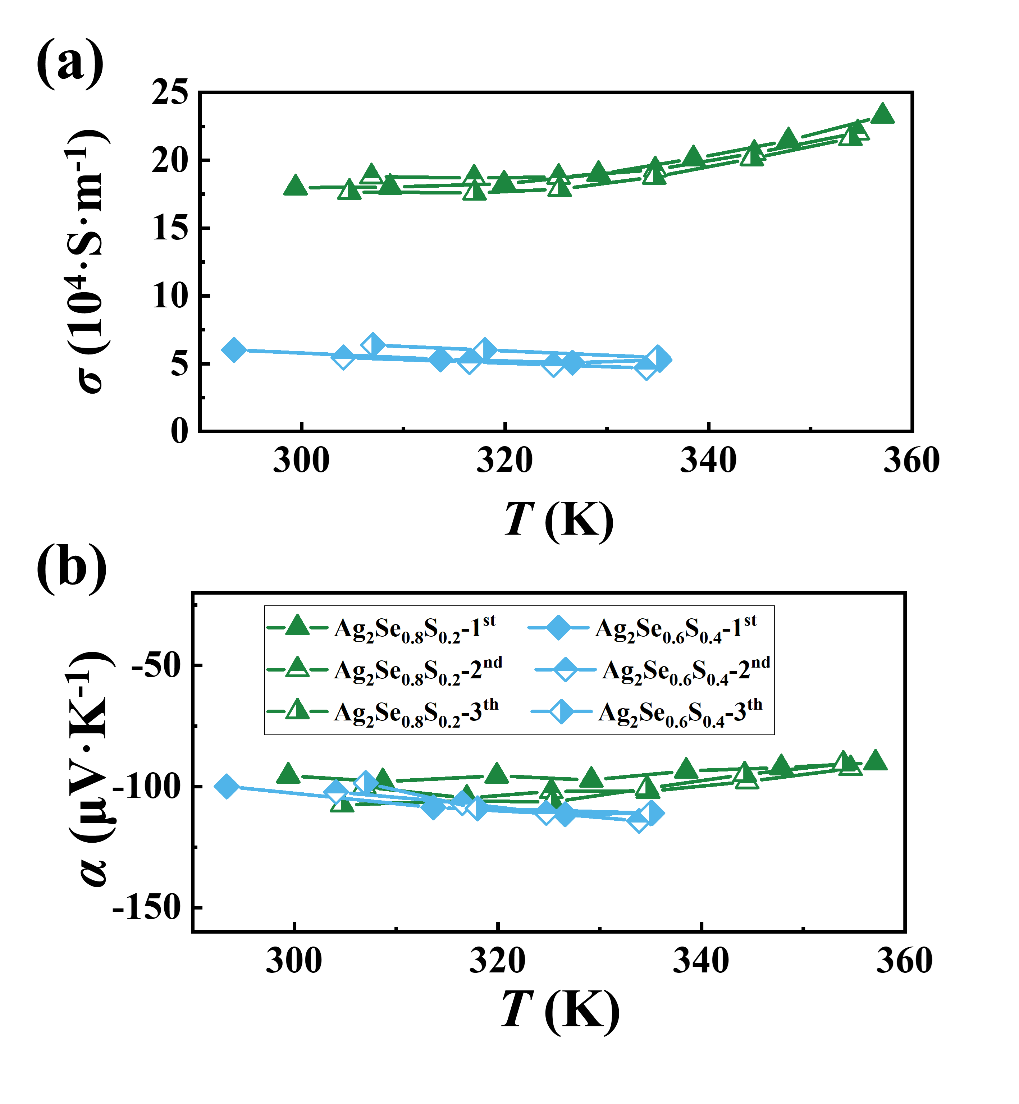


**Fig. S2** **(a)** Electrical conductivity and **(b)** Seebeck coefficient for three batches of Ag_2_Se_0.8_S_0.2_ and Ag_2_Se_0.6_S_0.4_.

**Table SI. Thermoelectric properties of Ag_2_Se_1-_*_x_*S*_x_* samples.** Room temperature Hall carrier concentration (*n_H_*), carrier mobility (*μ_H_*), electrical conductivity (*σ*), Seebeck coefficient (*S*), power factor (*PF*), TE figure of merit(*zT*), thermal conductivity (*κ*), and lattice thermal conductivity (*κ_L_*) of Ag_2_Se_1-_*_x_*S*_x_* (*x* = 0, 0.1, 0.2, 0.3, 0.4, and 0.45).

| **Composition** | **Ag_2_Se** | **Ag_2_Se_0.9_S_0.1_** | **Ag_2_Se_0.8_S_0.2_** | **Ag_2_Se_0.7_S_0.3_** | **Ag_2_Se_0.6_S_0.4_** | **Ag_2_Se_0.55_S_0.45_** |
| --- | --- | --- | --- | --- | --- | --- |
| ***n_H_* (cm^-3^)** | 6.5×10^18^ | 7.2×10^18^ | 1.1×10^19^ | 8.5×10^18^ | 9.3×10^18^ | 7.3×10^18^ |
| ***μ_H_***  **(cm^2^·V^-1^·s^-1^)** | 1337 | 865 | 995 | 961 | 404 | 309 |
| ***σ* (S·m^-1^)** | 1.4×10^5^ | 1.0×10^5^ | 1.8×10^5^ | 1.3×10^5^ | 6.0×10^4^ | 3.6×10^4^ |
| ***S* (μV·K^-1^)** | -128 | -140 | -96 | -104 | -100 | -119 |
| ***PF***  **(μW·cm^-1^·K^-2^)** | 22.5 | 19.6 | 16.4 | 14.0 | 6.0 | 5.2 |
| ***κ***  **(W·m^-1^·K^-1^)** | 1.1 | 1.0 | 1.4 | 0.94 | 0.70 | 0.65 |
| ***κ_L_***  **(W·m^-1^·K^-1^)** | 0.34 | 0.48 | 0.37 | 0.20 | 0.36 | 0.31 |
| ***zT*** | 0.61 | 0.58 | 0.35 | 0.44 | 0.26 | 0.24 |
